# Helminth Infection and Eosinophilia and the Risk of *Plasmodium falciparum* Malaria in 1- to 6-Year-Old Children in a Malaria Endemic Area

**DOI:** 10.1371/journal.pntd.0000164

**Published:** 2008-02-06

**Authors:** Philip Bejon, Tabitha W. Mwangi, Brett Lowe, Norbert Peshu, Adrian V. S. Hill, Kevin Marsh

**Affiliations:** 1 Kenya Medical Research Institute, Centre for Geographical Medicine Research (Coast), Kilifi, Kenya; 2 Centre for Clinical Vaccinology and Tropical Medicine, University of Oxford, Oxford, United Kingdom; 3 Wellcome Trust Centre for Human Genetics, University of Oxford, Oxford, United Kingdom; 4 Nuffield Department of Clinical Medicine, Oxford University, John Radcliffe Hospital, Oxford, United Kingdom; The University of Queensland, Australia

## Abstract

**Background:**

Helminth infection is common in malaria endemic areas, and an interaction between the two would be of considerable public health importance. Animal models suggest that helminth infections may increase susceptibility to malaria, but epidemiological data has been limited and contradictory.

**Methodology/Principal Findings:**

In a vaccine trial, we studied 387 one- to six-year-old children for the effect of helminth infections on febrile *Plasmodium falciparum* malaria episodes. Gastrointestinal helminth infection and eosinophilia were prevalent (25% and 50% respectively), but did not influence susceptibility to malaria. Hazard ratios were 1 for gastrointestinal helminth infection (95% CI 0.6–1.6) and 0.85 and 0.85 for mild and marked eosinophilia, respectively (95% CI 0.56–1.76 and 0.69–1.96). Incident rate ratios for multiple episodes were 0.83 for gastro-intestinal helminth infection (95% CI 0.5–1.33) and 0.86 and 0.98 for mild and marked eosinophilia (95% CI 0.5–1.4 and 0.6–1.5).

**Conclusions/Significance:**

There was no evidence that infection with gastrointestinal helminths or urinary schistosomiasis increased susceptibility to *Plasmodium falciparum* malaria in this study. Larger studies including populations with a greater prevalence of helminth infection should be undertaken.

## Author Summary

Malaria infection and other parasitic infections are widespread in developing countries. There is evidence from some studies that intestinal worm infections may increase the risk of developing febrile malaria. However, the evidence is mixed, and some studies have found no effect or even protective effects. A vaccine trial was recently conducted to assess the efficacy of a candidate malaria vaccine. Episodes of malaria were monitored. The vaccine was not protective, but data was also recorded on the prevalence of worm infections. The rates of febrile malaria did not seem to vary according to worm infection in this study. However, because of the relatively low prevalence of worm infection, the study did not have high power. Given the conflicting findings in the literature, and the potential for the effect of worm infection to vary geographically, it is important that larger, definitive studies are conducted, since even quite small effects might be important for global public health.

## Introduction

Helminth and malaria endemic areas frequently coincide, and an interaction between the two would be of considerable public health importance [1],[2]. Animal models suggest that helminth infections may alter susceptibility to malaria, although the results are conflicting [3],[4]. In studies conducted among malaria endemic populations in Africa, helminths have been reported to increase susceptibility to clinical malaria [5],[6], reduce the risk [7] or make no difference [8]. Studies in Thailand suggest helminths might increase the risk of non-severe malaria, but reduce the risk of cerebral malaria [9],[10].

The Th2 cytokine milieu induced by helminth infection is thought to drive the antibody response of malaria co-infected individuals towards the production of non-cytophilic subclasses (IgG2, IgG4, and IgM), whereas protection against malaria is associated with the presence of the IgG1 and IgG3 cytophilic subclasses [11]. The cytokine milieu could also favour either pro- or anti- inflammatory reactions during malaria infection [12].

We recently conducted a randomized controlled trial of a T cell inducing vaccine in a malaria endemic area. 387 immunised children were monitored for episodes of malaria for 9 months after receiving either an experimental T cell inducing vaccine or a control (rabies) vaccination [13]. Since helminth infection might be a covariate for risk of febrile malaria, and might interfere with vaccine efficacy [11], we prospectively obtained stool and urine samples for microscopy on all children enrolled into the trial, and measured peripheral eosinophilia. Here, we study the effect of helminth co-infections on the incidence of malaria.

## Methods

### Study participants

405 children, aged 1 to 6 years (inclusive), were randomized for either an experimental prime boost malaria vaccine or control vaccination (rabies). The trial was assigned registration number ISRCTN88335123 with the International Standard Randomized Controlled Trial Number Register (http://www.controlled-trials.com/isrctn/trial/. The study was performed with the permission of KEMRI National Ethics Committees, and COREC, the NHS Central Office for Research Ethics Committees. The children were healthy, and resident in the study area. The study area was limited to Junju sub-location in Kilifi district, Kenya. The entomological inoculation rate (EIR) is 22–53 infective bites/person/year [14]. Malaria transmission continues all year, with two seasonal peaks. The process of randomization and vaccination is detailed elsewhere [13]. Of these 405 children, follow-up visits were completed for 387 children, among whom 122 children had one or more episodes of *Plasmodium falciparum* malaria. Treatment with mebendazole and praziquantel was available in local dispensaries, but drug use was monitored among the study participants for the duration of the study by locally-based field workers.

### Samples

Eosinophilia was measured in blood taken one week post-vaccination (early May 2005). Single urine and stool specimens collected for microscopy in January 2006. Full blood counts were successfully analysed on 347 of the 387 children, and stool and urine samples collected for 315 and 294 children, respectively.

### Monitoring for malaria episodes

Children were seen weekly by field workers, and blood films made when the temperature was ≥37.5°C. Field workers lived in the study area, and parents brought their children for assessment between regular weekly visits if the child developed fever. Treatment for episodes of malaria was with the Government of Kenya recommended first line treatment, artemether-lumefantrine. In analysis, a threshold of 2,500 asexual parasites of *P falciparum* per µl was used to determine febrile malaria. This threshold was derived in previous studies in Kilifi for use in children above one year of age [15].

### Laboratory procedures

Blood films were examined in duplicate by two microscopists, and examined a third time if there was a discrepancy. Eosinophil counts were measured by a COULTER® Ac·T™ 5diff CP. Wet microscopy was conducted on mid-morning terminal urine samples and stool.

Children who were positive for schistosomiasis or gastrointestinal helminth infection were given praziquantel (40 mg per kg) or mebendazole (100mg twice daily for 3 days), respectively. Vaccination was complete by day 90, and treatment was given on day 150. All analysis is conducted on pre-treatment data.

### Analysis

The primary analysis was a log rank test comparing the time to the first or only episode of malaria between worm infected and uninfected children (unadjusted). The hazard ratio and 95% confidence interval was also estimated by Cox's regression, adjusted for age group, ITN (insecticide treated net) use and village. Age group was a categorical variable with three levels (1–2 years old, 2–5 years old, 5–6 years old). Village had 5 levels. ITN use was defined as sleeping every night under a treated net, which had less than three holes into which a finger could comfortably fit. The vaccination was not efficacious [13], and since adjusting for vaccine allocation made no difference to the results, the analysis presented here is unadjusted.

Poisson regression was used to estimate the incidence rate ratio taking into account all malaria episodes, adjusted for the same covariates. A period of 28 days after each malaria episode was deducted from the person time at risk.

## Results

### Gastrointestinal helminth infection

Stool samples were examined from 326 children. 25% were positive for ova indicating gastrointestinal worm infection. The three most common infections were *Ascaris lumbricoides* (18%), *Trichuris trichiura* (4.5%) and hookworm ova (3%).

**Figure 1:**
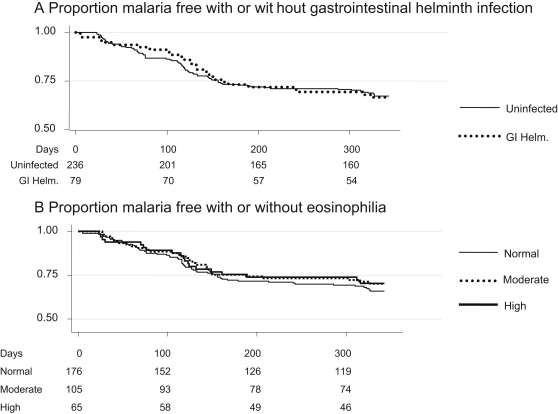
The probability of remaining free of clinical malaria is plotted over the 9 months of monitoring. Numbers of children at risk are given below the Kaplan Meier plots. Both plots use an endpoint of >2,500 parasites per microlitre and fever. Plot a) compares the probability of remaining free of clinical malaria for children with gastrointestinal helminth infection (GI Helm.) and uninfected children. Plot b) compares the probability of remaining free of clinical malaria for children with normal eosinophil counts (below 0.5×10^6^ per ml), mild eosinophilia (0.5–1×10^6^/ml) or high eosinophilia (above 1×10^6^/ml). The survival curves are not significantly different by unadjusted log-rank testing (p = 0.98 for gastrointestinal worm infection, p = 0.71 for eosinophilia).

The Kaplan Meier (KM) plot (Figure 1) of clinical malaria episodes (fever ≥37.5°C and parasitaemia >2,500 per µl) shows similar rates for children with and without gastro-intestinal helminth infections (p = 0.98). Children with positive stool samples were treated soon after day 150 with mebendazole.

**Figure 2:**
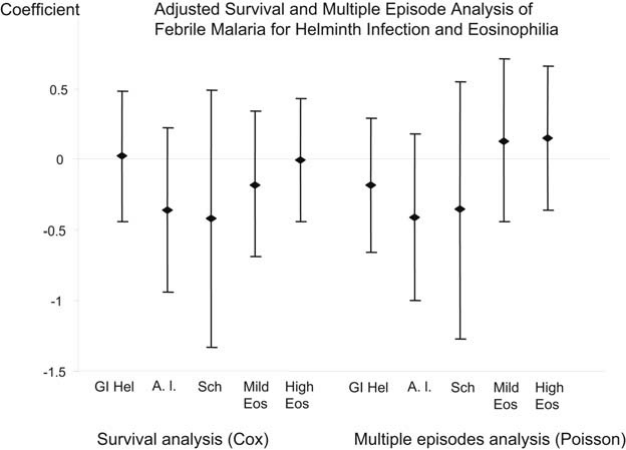
The coefficients from cox regression models (left) and the coefficients from poisson regression models (right) adjusted for the same covariates are displayed for the effect of gastrointestinal helminth infection (GI Hel), *A. lumbricoides* infection (A. l.), *S. haematobium* (Sch), mild eosinophilia (Mild Eos) and marked eosinophilia (High Eos), adjusted for age, village and ITN use.

Further modelling of the hazard and of multiple episodes (Figure 2) excluded the period after treatment. In a Cox regression model, adjusted for age, village and ITN use, the modelled hazard ratio (HR) for first episode of malaria was 1.01 (95% CI 0.64–1.57, p = 0.98). In a Poisson regression model for the number of malaria episodes, the incidence rate ratio (IRR) was 0.83 (95% CI 0.51–1.33, p = 0.45). The risk associated with *A. lumbricoides* infection alone was also considered. The HR for first episode of malaria was 0.65 (95% CI 0.39–1.25, p = 0.23) and in Poisson regression, the IRR was 0.67 (95% CI 0.37–1.2, p = 0.18).

### Urinary schistosomiasis

Urine samples were examined from 294 children. Only 24 children (8%) had eggs of *Schistosoma haematobium* in urine, as identified by microscopy. The confidence intervals of estimates of clinical malaria risk among children with urinary schistosomiasis are therefore wide, and so data on the survival function is not shown in detail. The adjusted HR was 0.55 (95% CI 0.23–1.38, p = 0.8) and the IRR was 0.69 (95% CI 0.28–1.73, p = 0.2. This is shown in Figure 2. Schistosomiasis and gastrointestinal helminth infection were not associated (χ = 0.53, p = 0.46) and only 7 children had dual infections.

### Eosinophilia

Eosinophil counts were available from 347 children. 19% had highly elevated counts (>1×10^6^/ml) and 31% had mildly elevated counts (>0.5×10^6^/ml). Peripheral blood eosinophil counts were conducted as a more sensitive marker of parasitic infection than microscopy. Neither Kaplan Meier plots (Figure 1, p = 0.71) nor adjusted estimates of risk (Figure 2) showed significant variations in malaria episodes by eosinophil count. Mild eosinophilia was associated with a HR of 0.85 (95% CI 0.51–1.43, p = 0.55) and an IRR of 0.86 (95% CI 0.51–1.4, p = 0.6). High eosinophilia was associated with a HR = 0.85 (95% CI 0.69–1.96, p = 0.47) and an IRR of 0.98 (95% CI 0.63–1.5, p = 0.57).

### Age prevalence of helminth infections

Schistosomiasis was seen in 0% of 1–2 year olds, 8% of 2–5 year olds and 14% of 5–7 year olds (p = 0.02), and gastrointestinal worm infections were seen in 11% of 1–2 year old, 25% of 2–5 year olds and 36% of 5–7 year olds (p = 0.003). Interestingly, eosinophilia became less frequent with age, from 63% of 1–2 year olds to 50% of 2–5 year olds and 42% of 5–7 year olds (p = 0.03).

### Anaemia and helminth infections

The average haemoglobin among children with gastrointestinal helminth infection was 10.47 g/dl (95% CI 10.2–10.8) and 10.21 g/dl (95% CI 10–10.4) among uninfected children, p = 0.23. For children with schistovuria the average haemoglobin was 10.83 g/dl (95% CI 10.3–11.4) and 10.33 g/dl (95% CI 10.1–10.5) among uninfected children, p = 0.16.

## Discussion

The data presented here were acquired during a trial designed primarily to examine vaccine efficacy. Children with gastrointestinal parasites or urinary schistosomiasis were treated with mebendazole or praziquantel, respectively. However, treatment was delayed for 150 days after screening, and the majority of malaria episodes (25% of children) occurred before the children were treated. Only 6% of children had a malaria episode after treatment (which coincided with the end of the high transmission season). We did not identify altered susceptibility to malaria associated with helminth infection, determined either by direct microscopy or inferred by peripheral eosinophilia.

There were similar findings in Uganda [8] where soil-transmitted helminths were not associated with an increased risk of febrile malaria. In Senegal, other cohort studies have suggested that schistosomiasis increases the risk of febrile malaria [6], and hospital-based case-control studies suggest both schistosomiasis and *A. lumbricoides* infection are risk factors for hospital admission with malaria [16]. However, a cohort study in Mali using weekly surveillance suggested that schistosome infection may actually protect from malaria [7]. Other studies suggesting that helminth infection might be protective relate to Thai adults, conducted by a group using case-control studies to examine severe disease [9], [17]–[19]. However, this group also conducted one cohort study examining febrile disease, where susceptibility was conferred by intestinal helminth infection [20]. Recent reviews of the evidence appear to focus on the possibility that helminth infection might increase susceptibility to malaria [1],[2],[11]. Of the cohort studies, the sample sizes were 80 [5], 511 [6], 654 [7] and 435 [8]. The prevalence of helminth infection was 16% [5], 67% [6], 25% [7] and 17–47% [8]. Our study with 387 children, and prevalence of 25%, 50% and 8% for intestinal infection, eosinophilia and schistosomiasis, respectively, is therefore quite comparable in terms of power.

Microscopy was only performed on a single specimen in our study, and we did not use concentration techniques. This would have missed lighter infections. This misclassification may have introduced bias against detecting an association. In previous studies that identified an increased risk of malaria in schistosome-infected children, multiple specimens were examined, although the greatest increase in risk was associated with heavier infections [6], which are not likely to have been missed by a single examination. Studies that report a positive association with soil-transmitted helminths did not depend on examining multiple specimens [5],[16], and even in a low prevalence area with light infections, the yield from repeated examinations is low [21]. The use of artesunate further complicates our assessment. This appears to result in a reduction in a relatively rapid reduction in egg production among infected children [22], and it is unclear how rapidly this might alter host susceptibility to malaria. Our primary analysis of gastrointestinal infection did not differentiate on the basis of species, but secondary analysis that was restricted to *A. lumbricoides* infection did not show a tendency towards increased risk of malaria in infected children either.

The low prevalence of helminth infection limits the power of the study, but by how much? This can be judged by the confidence intervals associated with the estimates of efficacy, from power calculations, and from inspecting the KM plots. The CIs around the estimates of risk are narrower than a halving or doubling of risk in each analysis conducted. The narrowest CIs are those derived for all gastrointestinal helminth infections and eosinophilia, followed by *A. lumbricoides* infection alone, with CIs for *Schistosoma haematobium* the widest. The tendency is in the direction of decreased rather than increased susceptibility, so the possible increases in risk that could have been missed are not large for gastrointestinal helminth infections and eosinophilia, but quite large effects of schistosome infection might have been missed. The primary analysis was log rank testing of time to event. Assuming a constant effect over time, with the prevalence of gastrointestinal helminth infection at 25%, then a log rank analysis had 80% power to detect a 70% increase or 50% decrease in risk due to helminth infection. Power was very low for schistosomiasis, with 80% power to detect a 110% increase or 90% decrease, but 80% power to detect an 80% increase or 60% decrease for *A. lumbricoides* infection alone. However, as it turned out, the upper limits of the confidence intervals measured suggest that 60–80% increases in risk are unlikely, although greater decreases in risk have not been ruled out. Previous studies have suggested a doubling in risk of febrile malaria in schistosome-infected children [6], and 64% from soil-transmitted helminths [5]. The increased risk seen secondary to schistosomiasis was seen only in the heavily infected children, and our study did not differentiate heavy from light infections.

On inspection of the KM plots, there is no marked separation of the helminth-infected and uninfected at any stage. Between 50 and 125 days, helminth-infected children have acquired slightly fewer cases of febrile malaria, but this difference is slight, and the lines converge by 150 days. After 150 days there is a slight reduction in the number of children with febrile malaria among those with moderate and high eosinophil counts. This suggests that further studies to examine the impact of helminth infection in this population would need to be considerably larger.

The results of individual studies may be dependant on the transmission intensity of both worm and Plasmoidium infections. In our study, the moderately high malaria transmission meant that significant immunity had been acquired by children aged 5–7 compared with 1–2 year olds (HR 0.24, 95% CI 0.1–0.54, p = 0.001). However, worm infection is more frequent in the older age groups, and the interaction between worms and malaria may depend critically on being exposed to helminth infections while immunity to malaria is being acquired. We did not measure intensity of infection in this study, and it is possible that among older children, with more intense worm infections, there might have been a relationship between helminth infection and malaria. However, these children would have a low incidence of malaria. Previous studies identifying increased susceptibility to malaria among helminth infection have been conducted in settings of more prevalent helminth infection.

50% of children had either mild or high eosinophilia, compared with a prevalence of 7% for urinary schistosomiasis and 25% for gastrointestinal helminth infection. The higher prevalence may reflect infections missed by microscopy, but also could be due to filarial infection (which we did not examine for). *Strongyloides stercoralis* infection is also associated with eosinophilia, and would not have been identified by the stool examination conducted in this study. Eosinophilia is closely associated with the TH2 cytokine responses which might result in the increased susceptibility to malaria [23], and might therefore have been expected to be a more discriminatory marker. Eosinophilia was not associated with altered rates of malaria in this study. However, the potentially deleterious responses that can be induced by helminths include TH1 and regulatory responses, of which eosinophilia would not be a marker. We found that eosinophilia became less prevalent with age, despite the prevalence of helminth infection rising, suggesting that eosinophilia becomes suppressed with chronic helminth infection. As well as immunological mechanisms [24],[25], it has been hypothesised that helminth induced anaemia induces susceptibility to malaria [24], although other data suggest that iron deficiency protects against malaria [26]. In any case, helminth infected children in our study were not significantly more anaemic.

This study suggests that in a cohort of one to six year old children in a moderately high malaria transmission area are not at increased risk of malaria from concurrent helminth infection, as evidenced either by microscopy or by eosinophilia. In our study, the prevalence on helminth infection was relatively low, and heavy infections were probably infrequent. This reflects the age of the children enrolled. However, it would not have been practical to study older children at this transmission intensity, since immunity against febrile disease is acquired rapidly (the HR among five to six year old children was 0.29 (95% CI 0.15–0.58). In areas with lower malaria transmission, older children may retain susceptibility to malaria.

What further studies should be conducted? An interaction between helminth infection and malaria is of considerable public health importance. If present only at lower transmission intensities, this would still account for a very significant number of infections globally [27], and even a slight impact on high transmission areas might still be significant [2]. That we have not identified an effect in the present study does not exclude a significant effect, given the caveats associated with the methodology and sample size. Further studies will need more detailed assessments of individual children as well as a greater sample size. To overcome the caveats identified in this study, multiple examinations of quantified stool specimens will be required during the period of follow-up, multiple geographical areas and a wider age group should be included, and attention should be given to filarial infection, *Strongyloides stercoralis* and eosinophilia. Only a large and detailed study of this nature will satisfy concerns about multiple infections, changing infection status of individual children, and increased susceptibility depending critically on worm burden and age of acquisition of immunity to malaria.
